# Neural network of bipolar disorder: Toward integration of neuroimaging and neurocircuit-based treatment strategies

**DOI:** 10.1038/s41398-022-01917-x

**Published:** 2022-04-05

**Authors:** Bo Bi, Dongfang Che, Yuyin Bai

**Affiliations:** 1 Department of Clinical Psychology, The Eighth Affiliated Hospital, Sun Yat-Sen University, Shenzhen, China; 2 Neurosurgery department, Shenzhen Children’s Hospital, Shenzhen, China

**Keywords:** Neuroscience, Pathogenesis

## Abstract

Bipolar disorder (BD) is a complex psychiatric disorder characterized by dysfunctions in three domains including emotional processing, cognitive processing, and psychomotor dimensions. However, the neural underpinnings underlying these clinical profiles are not well understood. Based on the reported data, we hypothesized that (i) the core neuropathology in BD is damage in fronto-limbic network, which is associated with emotional dysfunction; (ii) changes in intrinsic brain network, such as sensorimotor network, salience network, default-mode network, central executive network are associated with impaired cognition function; and (iii) beyond the dopaminergic-driven basal ganglia-thalamo-cortical motor circuit modulated by other neurotransmitter systems, such as serotonin (subcortical–cortical modulation), the sensorimotor network and related motor function modulated by other non-motor networks such as the default-mode network are involved in psychomotor function. In this review, we propose a neurocircuit-based clinical characteristics and taxonomy to guide the treatment of BD. We draw on findings from neuropsychological and neuroimaging studies in BD and link variations in these clinical profiles to underlying neurocircuit dysfunctions. We consider pharmacological, psychotherapy, and neuromodulatory treatments that could target those specific neurocircuit dysfunctions in BD. Finally, it is suggested that the methods of testing the neurocircuit-based taxonomy and important limitations to this approach should be considered in future.

## Introduction

Bipolar disorder (BD) is a complex psychiatric disorder characterized by severe mood fluctuations associated with dysfunctional cognition that often persist throughout the entire life of patients and abnormal psychomotor activity [1, 2]. The heterogeneous phenomenological dimensions of the various bipolar symptoms can be systematized and traced back to combined changes of affectivity, cognition, and psychomotricity. With the advances in neuroimaging technology, there are increasing opportunities to understand the causes, mechanisms, and treatment of BD. With mounting evidence suggesting that the abnormalities between cortical and subcortical regions associated with emotion, cognition, and psychomotor activity are likely risk markers for BD [3–5].

BD has traditionally been associated with dysfunction in the fronto-limbic neural circuits underlying emotional regulation, as well as hypersensitivity in the fronto-striatal neural circuits responsible for rewarding processing, although it is now recognized that alterations in distinct large-scale networks seems to play a fundamental role in the phenomenological dimensions, including affective, cognition, and motor functions [6–10]. Most of neurobiological models of BD suggest a dysfunction of the dorsal and ventral systems, composed, respectively, of (i) the prefrontal cortex (PFC), anterior cingulate cortex (ACC) and the hippocampus and (ii) the insula, amygdala, and ventral striatum [6, 11]. The emotional regulation and mood liability could be due to alterations in these two partially overlapping ventral prefrontal systems, which may result in a loss of homeostasis in emotional processing [12].

Functional neuroimaging research has revealed intrinsic brain activities, the functional architecture as organized in distinct brain networks in accordance to connectivity patterns, and related setting of input/output processing involve in the psychopathology of affectivity, psychomotricity, and thought in BD [13]. Within the last decade, evidence from neuroimaging has highlighted four related largescale intrinsic brain networks that are implicated in a range of cognitive processes and are considered to be domain-general functional networks that support many cognitive function, i.e. the default mode network (DMN), the salience network (SN), the cognitive control network (CEN), and the sensorimotor network (SMN) [14–18]. Abnormalities in these aspects likely are associated with different neurocircuits that interact with each other to generate the complex BD phenotype.

The expression of psychomotor dimensions of BD is traceable to modulation of motor cortex and motor network by non-motor cortical networks, i.e., default-mode network and sensory networks. Additionally, the dopamine-driven basal ganglia-thalamo-cortical motor circuit modulated by other monoaminergic neurotransmitter systems such as serotonin (subcortical–cortical modulation) is also involved in psychomotor mechanisms [19]. Based on these three dimensions that are constructs derived from fundamental phenomenological components, which can be linked to neurobiological systems and fostered by the recent advancement of sophisticated neuroimaging techniques, recent neuroimaging studies of BD demonstrate abnormalities in neural circuits supporting emotional response and regulation, and cognition processing, and producing and controlling motor behavior and the integration of sensory information. Therapeutic approaches based on the patterns of alterations in these neurocircuits and clinical factors varying across individuals could improve treatment efficacy and guide clinical practice. However, it remains unclear how these different clinical profiles are associated with specific pathophysiological mechanisms involved, in particularly the brain regions and networks that ultimately support these functions or their usefulness in guiding treatment selection.

In this review, we enlarge on previous models of the neurocircuitry of BD to propose a heuristic neurocircuit-based taxonomy, which could be used to guide the diagnosis and treatment of the disorder in future. We present hypothetical links between underlying neurocircuit dysfunctions and the three domains of clinical presentation in BD and discuss how treatments that more specifically target these circuits may benefit patients and improve therapeutic response. We suggest ways in which this neurocircuit-based taxonomy could be tested in future research to support or refute our proposed links between aspects of clinical phenomenology across different mood states, specific neurocircuit alterations, and treatment methods. Likewise, the neurocircuits are not as segregated as they may appear in the following sections and highly interactive, and the neurocircuits are certainly more complex and not limited to the neurocognitive and behavioral alterations we describe here.

## Emotional regulation and processing dysfunctions of neural network in BD

Emotional hyper-reactivity and mood instability have a detrimental impact on functioning, relapses, and suicide attempts in BD patients. Excessive reactivity to negative events might be a core dimension of BD and could represent an endophenotype during both manic and mixed states in comparison to patients in remitted phases [7, 20]. Research in the field of affective neuroscience conducting neuroimaging studies sheds light on correlations between activities in different brain regions, and the findings can provide insights on the role of different regions in emotion regulation. In the past few decades, the increasing neuroimaging studies have demonstrated that the frontal-limbic connectivity abnormally is a promising biomarker of the pathophysiology and maintenance of BD, which can be considered as the core of affectivity [12, 21].

**Fig. 1:**
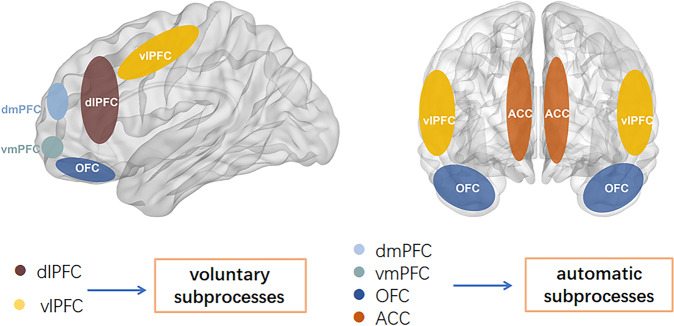
Voluntary subprocesses and automatic subprocesses. The lateral prefrontal cortical regions (dlPFC and vlPFC), which develop relatively late and are involved in higher executive functions may subserve voluntary subprocesses. The automatic subprocess includes the area of dmPFC, vmPFC, OFC, ACC, and are involved especially in the control of emotional behaviors. dlPFC dorsolateral prefrontal cortex, vlPFC ventrolateral prefrontal cortex, dmPFC dorsomedial prefrontal cortex, vmPFC ventromedial prefrontal cortex, OFC orbitofrontal cortex, ACC anterior cingulate cortex.

We will first provide an overview of the prefrontal cortical regions including the anatomy and the fronto-limbic functional connectivity that have been implicated in emotional processing. The functional localization and division of the human prefrontal cortex are orbitofrontal cortex (OFC), dorsomedial prefrontal cortex (dmPFC), dorsolateral prefrontal cortex (dlPFC), ventrolateral prefrontal cortex (vlPFC), and ACC [22]. The ventromedial prefrontal cortex (vmPFC) which develop relatively early comprises OFC, dmPFC, and ACC and are involved especially in the control of emotional behaviors, whereas the lateral prefrontal cortical regions (dlPFC and vlPFC) develop relatively late and are involved in higher executive functions [23]. The former neural system may be involved in automatic subprocesses, whereas the latter neural system may subserve voluntary subprocesses (Fig. 1). The combination of the functional and structural abnormalities in neural systems implicated in voluntary and automatic subprocesses of emotion regulation may underlie the mood instability of BD [24]. Those regions are the most densely connected with the amygdala and other subcortical limbic and paralimbic regions [25].

**Fig. 2:**
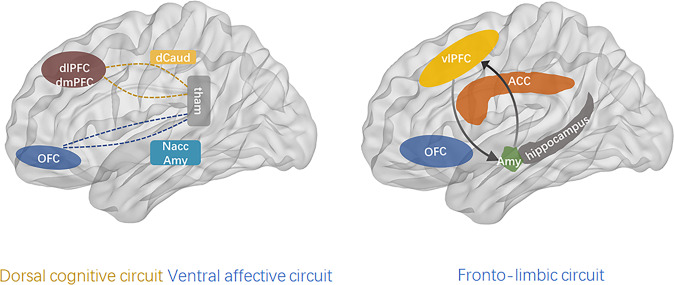
The dorsal cognitive circuit includes the dorsolateral prefrontal cortex (dlPFC), dorsomedial prefrontal cortex (dmPFC), dorsal caudate (dCaud), and thalamus, and is involved in executive functions (e.g., working memory, planning) and emotion regulation. The ventral affective circuit includes the orbitofrontal cortex (OFC), ventral striatum (particularly the nucleus accumbens (NAcc)), and the thalamus. This circuit is crucially involved in reward functions, which are largely mediated by dopaminergic signaling. The fronto-limbic circuit mainly includes the prefrontal cortex and amygdala, and hippocampus. These brain regions are structurally and functionally connected with each other to form a network that generates emotional responses and evaluates whether those responses are appropriate or require regulation. vlPFC ventrolateral prefrontal cortex, Amy amygdala, ACC anterior cingulate cortex. dmPFC dorsomedial prefrontal cortex, vmPFC ventromedial prefrontal cortex, OFC orbitofrontal cortex, ACC anterior cingulate cortex. dlPFC dorsolateral prefrontal cortex, vlPFC ventrolateral prefrontal cortex, dCaud dorsal caudate, tham thalamus, NAcc accumbens, Amy amygdala.

The fronto-limbic circuit includes subcortical and cortical brain regions involved in generating emotional responses (amygdala and nucleus accumbens) and evaluating whether those responses are appropriate or require regulation (vmPFC) [26]. The vmPFC regions relate to each other to form a network structurally and functionally that generates emotional responses and evaluates whether those responses are appropriate or require regulation (vmPFC). The ventral affective circuit is also connected with the hippocampus and the dorsal cognitive circuits, which includes dlPFC, dmPFC, dorsal caudate, and thalamus, and is involved in executive functions (e.g., working memory, planning) as well as emotional regulation (Fig. 2).

It is hypothesized that the abnormal emotional ventral system and hypoactive dorsal system result in a loss of homeostasis in emotional processing, which may contribute to mood liability in BD disorder. This ventral network is involved in identifying salient emotional stimuli and in mediating autonomic responses; while the dorsal system is usually involved in selective attention, planning and explicit regulation of emotional states, with its hypoactivation being underpinning of the unstable cognitive control of emotions and affect in BD [27]. The deficits of emotional processing and regulation in BD involve decreased activity of vlPFC and increased acvity of amygdala, striatal, and mPFC [28, 29]. These abnormalities may be predominately within the left vmPFC regions implicated in automatic emotion regulation, and neuroimaging studies have demonstrated greater subcortical limbic activity (including amygdala, ventral striatum, and hippocampus) to emotional stimuli in adult BD during mania, depression and euthymic, compared to healthy individuals. Patients with BD showed hypoactivation of vlPFC, which also persist in euthymia and might represent a trait feature of BD [30]. More recent work suggests that BD patients show reduced connectivity between the vlPFC, ACC, OFC, and limbic areas across mood states [31–33].

Moreover, dysregulation of the amygdala by PFC seems to be crucial to better understand the pathophysiology of the disease. The amygdala is an evolutionarily ancient critical area within the fronto-limbic system that is responsible for flight/fight responses to threats, and is hyperactive in mania, whereas contrasting results have been found during depression [34]. Systematic review on functional connectivity between the amygdala and its projecting areas highlights the essential role of the amygdala in attributing the stimuli’s valence, influencing emotional, and behavioral responses [35]. Several investigators observed increased left amygdala activation in the manic group during facial affect matching, while other groups reported similar findings in BD individuals during depression and remission and with other tasks. Table 1 for a summary of the fronto-limbic system results of functional magnetic resonance imaging emotion studies in bipolar.

**Table 1:** The fronto-limbic system results of fMRI emotion studies in bipolar.

Study	*N*	Paradigm	Method	Connections	Main findings
Versace et al. (2010)	E = 17 D = 14 HS = 24	Happy or sad faces	FC	Amy–OFC	For sadness: D > E > HS
					For happiness: D < HS
Brady et al. (2017)	M = 26 E = 10 BD = 10	*N*	rsfMRI	Amy–ACC	M < E
			FC	Amy–SMA	M > E
Townsend et al. (2013)	E = 26 HS = 26	Emotional task	PPI	Amy–VlPFC、ACC.	HS > E
Vizueta et al. (2012)	D = 21	Emotional task	FC	Amy–OFC	HS > D
	HS = 21			Amy–DLPFC	
Anticevic et al. (2013)	E = 68	*N*	Global brain connectivity and ROI	Amy–DLPFC	HS > E BD-1
	HS = 51			Amy–MPFC	E BD-2 > HS
Radaelli et al. (2014)	D = 52	Emotional task	Dynamic causal modeling	DLPFC–Amy	HS > D
	HS = 40				
Lippard et al. (2021)	M = 42	Emotional task	FC	ACC–Caudate	Non-R > HS
	R = 21			ACC–Amy	R > baseline
	HS = 41				
Mullin et al. (2012)	E = 22	Emotional task	Ganger causality	ACC–Amy	HS > E
	HS = 19				
Guo et al. (2020)	E = 16	Cognitive task	FOCA	OFC–Amy	E < HS
	HS = 16			SFG–Insula	
Hafeman et al. (2017)	BD = 22	Emotional task	FC	VLPFC–Amy	B > HS/ADHD
	ADHD = 30				
	HS = 26				

*BD* bipolar, *E* euthymic, *M* mania, *D* depression, *HS* healthy subjects, *FC* functional connectivity, *Amy* amygdala, *VLPFC* ventrolateral prefrontal cortex, *DLPFC* dorsolateral prefrontal cortex, *OFC* orbitofrontal cortex, *MPFC* medial prefrontal cortex, *ACC* anterior cingulate cortex, *ROI* region of interest, *PPI* psychophysiological interaction, *FOCA* four-dimensional consistency of local neural activities, *SFG* superior frontal gyrus, *ADHD* attention deficit hyperactivity disorder.

## Treatments targeting dysfunctional emotion regulation and processing in BD

Most of the treatment regimens of BD owe their discovery to combination of fortune, intellectual rigor, and the careful observation of human and animal phenotypes [36]. The understandings of neuroanatomical basis and pathophysiological underpinnings of BD are increasingly reflected in the treatments moving into clinical trial. Pharmacotherapy, cognitive-behavior intervention, and neuromodulation have been applied across all phases of the bipolar illness and are considered as evidence-based treatments for BD.

Current pharmacotherapy for BD is based on administration of mood stabilizers (lithium, valproate, carbamazepine, and lamotrigine) and increasingly, on the administration of second-generation antipsychotics. It is worth mentioning that lithium appeared to induce a rapid suppression of amygdala over-activation and to show substantial treatment response in BD. Results from neuroimaging studies in patients with BD treated with lithium support the hypothesis that enhancement of neuroplasticity is a potential mechanism underlying the therapeutic effects of lithium. Such studies have also revealed brain changes in both gray and white matter, especially in hippocampal volume [37, 38].

Currently, an increasing number of meta-analyses have evaluated the efficacy of cognitive-behavioral therapy (CBT) for BD [39–41]. CBT is based on the hypothesis that BD involves dysregulated cognitive reappraisal, which involves reconceptualization of the emotion-eliciting situations in a way that will decrease emotional intensity. The cognitive reappraisal aspect of CBT engages dorsal cognitive circuitry, i.e., the top-down dorsal prefrontal regions to downregulate overactivated fronto-limbic activity, which may benefit individuals with impaired emotion regulation (dorsal cognitive circuit dysfunction) [26]. CBT seems to be mediated by modulation of the amygdala-dlPFC connectivity, and these findings lead to the hypothesis that the disturbance in effective connectivity from the dlPFC to the amygdala while reappraising in BD patients is due to insufficient prefrontal control. CBT, although not explicitly addressed in treatment, has been shown to reduce emotional symptomatology [42].

Neuromodulation holds promise for a vast number of medically refractory neurological and psychiatric disorders and has been guided by advances in neuroimaging and neurophysiology, which had led to new strategies for better targeting, identification of optimal stimulation sites, and understanding of its therapeutic mechanisms. Repetitive transcranial magnetic stimulation (TMS) is a dominant noninvasive stimulation treatment that delivers short magnetic pulses via an electromagnetic coil and is operated to transiently stimulate or inhibit specific brain regions. TMS has had a growing impact on the treatment of unipolar depression and has already been approved by the FDA for use in treatment-resistant depression [43]. Although there is no clear evidence available to base upon for using TMS in treatment of BP, but it is readily advantageous in similarly leveraging for use in treatment-resistant bipolar depression.

A recent review on TMS for BD suggests the potential of repetitive TMS for reducing depressive symptoms [44]. It has been shown that the TMS protocol over the right dlPFC yields a particular benefit for mania, while over the left dlPFC leads to improvement in depressive symptoms [45]. The first meta-analysis of 19 randomized controlled trials of TMS in the treatment of acute bipolar depression in 181 patients suggests that TMS is efficacious, with overall treatment efficacy in BD comparable with in major depression disorder (MDD). Furthermore, the TMS treatment is safe with regard to treatment-emergent affective switches [46]. A preliminary study also showed that deep TMS is a potentially effective and well-tolerated add-on for pharmacotherapy in resistant bipolar depressed patients [47, 48]. The reviewed evidence collectively suggests the potential of TMS in treating BD in both depression and mania phase. However, it should be noted that there is no adequately powered trial conducted yet to further support the efficacy of TMS in BD. When compared with sham treatments, most RCTs found no significant differences in depression symptoms [49]. Paul et al. examined the effectiveness of sequential (to the right dlPFC and then the left dlPFC) bilateral repetitive transcranial magnetic stimulation in the treatment of bipolar depression and found there was no significant difference in mean reduction in depression rating scale scores or response rates between active and sham stimulation [50]. The clear diagnosis of bipolar in the depressive phase, a stable platform of medications (we suggest mood stabilizer and antipsychotics), and a personalized approach to TMS stimulation are essential and likely needed. Table 2 for summary of rTMS and tDCS clinical trials in BD.

**Table 2:** Summary of rTMS and tDCS clinical trials in bipolar disorder.

Study	*N*	Design	Mood episode	Sessions	Main findings
Grisaru et al. (1998)	16	Randomized study	Mania	10	YMRS ↓ CGI ↓
Tavares et al. (2017)	50	RCT	Depression	20	HAMD ↓
Fitzgerald et al. (2016)	49	RCT	Depression	20	N.S
Kazemi et al. (2016)	30	Randomized study	Depression	20	Bilateral > Unilateral
Hare et al. (2011)	19	Open study	Depression	20	HAMD ↓
Hu et al. (2016)	38	RCT	Depression	20	HAMD ↓ MADRS ↓
Dell’Osso et al. (2015)	33	Randomized study	Depression	20	HAMD ↓ MADRS ↓ CGI ↓
Kazemi et al. (2018)	20	Open study	Depression	10	Verbal memory↑ executive functions↑
Tortella et al. (2021)	59	Open study	Depression	10	N.S
Minichino et al. (2015)	21	Open study	Euthymic	15	Visuospatial memory↑ executive functions↑

*YMRS* young manic rating scale, *CGI* clinical global impression, *HAMD* Hamilton rating scale for *MADRS* Montgomery–Asberg depression rate scale *N.S.* not significant.

## Cognitive alterations of neural network in BD

Mounting evidence suggests BD is associated with common cognitive deficits, including executive function, memory, social cognition, and response timing [51–53]. A large body of neuroimaging research has focused on topological network analyses of brain circuit, suggesting that modular and hierarchical structural networks are particularly suited for the functional integration of local neuronal operations that underlie cognitive function [54]. Synchronous activity and antisynchronous activity between neural elements at rest reflects the physiological process underlying complex cognitive ability. The tightly interconnected brain networks have emerged as the fundamental, dynamically organized elements of human brain function, consisting of patterns of synchronized activity across different brain regions [55–57]. These intrinsic brain networks are composed of hubs involved in important cognitive functions such as behavioral regulation and attention control and has transdiagnostic features that can serve as markers of affective and psychotic pathologies [58]. How cognitive processing emerges from interacting elements within the entire brain can be more meaningfully probed using a network theory framework. Neuroimaging research has revealed that the intrinsic brain activity, which is organized in distinct large-scale networks has been implicated in a variety of cognitive functions that are commonly disrupted in BD [59].

Although the neurophysiological basis of cognition and emotion are typically studied independently, recent work in this field increasingly highlight evidence showing substantial overlap in brain regions and networks involved in both emotion and cognitive processes. Cognition‐emotion integration requires the dynamic coordination and flexible switching between four functional networks that are linked to key adaptive processed, including emotion regulation, goal-directed attentional control, decision making, and self-monitoring [60].

**Fig. 3:**
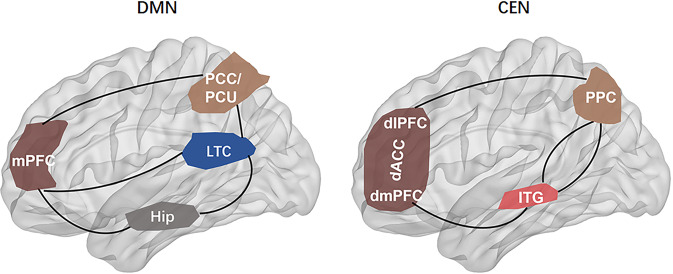
The default mode network extending from the prefrontal medial cortex (mPFC) to the posterior cingulate cortex (PCC), PCU precuneus cortex, lateral temporal cortex (LTC), temporal pole (TempP), hippocampus (Hip) is involved in, respectively, in multiple cognitive and affective functions, such as emotional processing, self-referential mental activity, mind wandering, recollection of experiences and possibly exerts a modulatory role during attentional demanding tasks. The central executive network (CEN), also known as the “cognitive control network, includes the dorsolateral prefrontal cortex (dlPFC), dorsal ACC, posterior parietal cortex, inferior temporal gyrus, and precentral gyrus, and is involved in attention-demanding cognitive tasks and shows increased activity in frontal and parietal regions associated with top-down modulation of attention and working memory tasks. mPFC medial prefrontal cortex, PCC posterior cingulate cortex, PCU precuneus cortex, LTC laterl temporal cortex, Hip hippocampus, dlPFC dorsolateral prefrontal cortex, dACC dorsal ACC, dmPFC dorsomedial prefrontal cortex, PPC posterior parietal cortex, ITG inferior temporal gyrus.

**Fig. 4:**
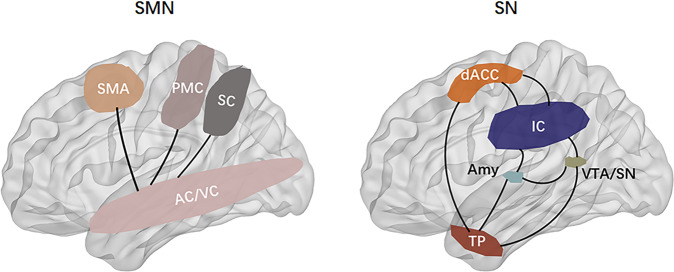
The sensorimotor network (SMN) mainly comprises the motor cortex (precentral gyrus, premotor, and supplementary motor cortex) and middle cingulate cortex, somatosensory cortex (postcentral gyrus and parietal cortex), auditory cortex (superior temporal gyrus), as well as visual cortex (occipital cortex and associated dorsal parietal and inferior temporal areas). The salience network (SN) consisting of the insular cortex, the dorsal ACC, the amygdala, and the temporal pole, is involved in interceptive awareness, task-set maintenance, and detection of salient stimuli from the environment. dACC dorsal ACC, IC insular cortex, Amy amygdala, TP temporal, VTA/SN ventral tegmental area/ substantia nigra. SMA supplementary motor area, PMC primary motor cortex, SC somatosensory cortex, IC insular cortex, AC auditory cortex, VC visual cortex.

**Fig. 5:**
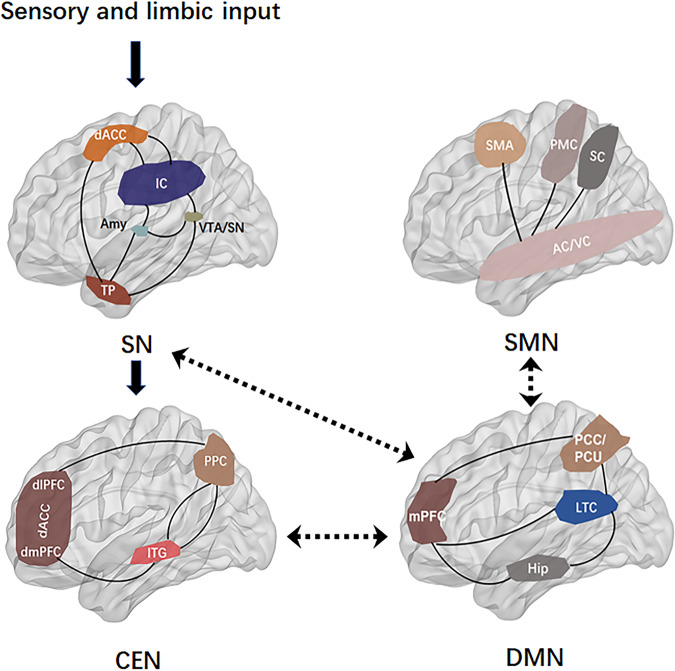
The salience network (SN) plays a central role in switching between the default mode network (DMN) and central executive network (CEN), and abnormalities of the SN could lead to weak salience mapping and give rise to dysfunctions of the CEN and DMN. The DMN/SMN balance was tilted toward the DMN in depression and toward the SMN in mania with bipolar disorder.

**Fig. 6:**
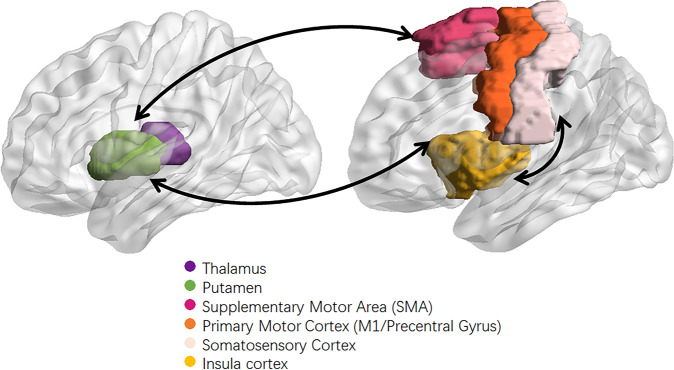
Sensorimotor network. The sensorimotor network (SMN) mainly comprises the motor cortex (precentral gyrus, premotor and supplementary motor cortex) and middle cingulate cortex, somatosensory cortex (postcentral gyrus and parietal cortex), auditory cortex (superior temporal gyrus), as well as visual cortex (occipital cortex and associated dorsal parietal and inferior temporal areas) and is involved in exteroceptive awareness and motor activity.

The DMN, also known as the “task-negative network”, was initially identified as brain regions showing consistently synchronized deactivation during tasks and prominent activation during rest [61] (Fig. 3). The CEN, also known as the “cognitive control network, includes the dlPFC, dorsal ACC, posterior parietal cortex, inferior temporal gyrus, and is involved in attention-demanding cognitive tasks and shows increased activity in frontal and parietal regions associated with top-down modulation of attention and working memory tasks [56, 62] (Fig. 3). The DMN and the CEN are often seen as opponent networks as the DMN is most active during rest, while CEN is most active during cognitive tasks. The SN typically consists of the fronto-insular cortex, the dorsal ACC, the amygdala, and the temporal pole, and is involved in interceptive awareness, task-set maintenance, and detection of salient stimuli from the environment [14] (Fig. 4). The SN plays a central role in switching between the DMN and CEN, and abnormalities of the SN could lead to weak salience mapping and give rise to dysfunctions of the CEN and DMN (Fig. 5). It has been reported that DMN and CEN act conversely, with the SN-mediating activity between the two networks, and both CEN and SN negatively regulate DMN function. The SMN displays coherent low-frequency (<0.1 Hz) activity fluctuations, which the sensory and motor/premotor cortical areas are reciprocally connected with the thalamus structurally and functionally [63] (Fig. 6).

Recently, evidence from fMRI studies point to aberrant activity of networks in BD as common neuronal correlates of cognitive impairment [64–67]. It has been shown that cognitive impairments arise from disruption of neuroplasticity and associated functional as well as structural changes in cognition-relevant neurocircuitries, with identified abnormalities in above four networks [8, 9, 68, 69]. The major finding indicates that BD is associated with alterations in both frontal and posterior DMN structures, mainly in the prefrontal, posterior cingulate and inferior parietal cortex [66]. Moreover, the hyperactivity of DMN has been clearly shown to ruminations and excessive internal (self-focused) thought, potentially playing a core role in depressive states. Martino et al. conducted a series of studies on BD investigating the different phases separately. It was found that the network balance in intrinsic brain activity was tilted toward the DMN in the depression phase but was shifted toward the SMN during mania [9]. The functional connectivity analyses revealed significantly stronger positive correlations between dlPFC and DMN (subgenual cingulate cortex) regions in asymptomatic patients with BD, suggesting BD patients may have greater difficulty switching from internally focused processing to task‐related processing in the presence of competing cognitive‐affective demands, compared to controls [70, 71]. Thus, the cognitive impairments in BD are likely caused by a failure to recruit key regions in the CEN to suppress task irrelevant DMN activity during cognitive performance [72].

In addition to decreased frontoparietal CEN recruitment, cognitive dysregulation in BD is also associated with disrupted SN recruitment [73]. A recent study has shown that bipolar depression is distinguished from both unipolar depression and healthy controls by significantly altered bilateral dorsal anterior insula functional connectivity to the left inferior parietal lobule (IPL), a key node of the frontoparietal CEN [74]. Another major finding is the cardinal role of the SN in BD disorder, characterized by a decrease in FC in several frontal and parietal regions and an increase in FC with the postcentral gyrus, precentral gyrus, temporal, and occipital regions. This conclusion is consistent with the meta-analysis suggesting the frontoparietal network may be the core common deficit in psychiatric disorders [75]. It has also been shown that cognitive impairment occurs across the psychosis spectrum and is associated with reduced cingulo-opercular network (CON), and subcortical network efficiency observed across the psychosis spectrum [76]. For example, a recent meta-analysis focused on the structural and functional brain abnormalities in unaffected relatives of individuals with BD. The results revealed hyperactivations in the fronto-striatal regions as well as hypoactivation in parietal regions during cognitive tasks, activation in the amygdala during emotional processing, and in the OFC during reward-related tasks [77]. In unaffected relatives, the right inferior frontal gyrus had greater volume and higher activation during cognitive tasks [77]. Regarding the preclinical studies investigating cognitive function in BD, we concluded the patients’ cognitive impairments may also be exacerbated by a failure to suppress neural activity in the DMN, most consistently the medial PFC and be associated with the aberrant encoding and working memory-related activity in the fronto-parietal, temporal regions during acute mood episodes and remission [78, 79]. A highly consistent finding was that aberrant functional connectivity within subcortical and PFC structures was linked with impaired? Working memory performance in BD [80, 81].

In BD, graph analysis studies indicate distinctive abnormalities of node-level and inter-hemispheric connectivity patterns, altered DMN dysconnectivity and decreased white matter organization of anterior limbic system tracts supported by reductions in efficiency and clustering connecting cingulate and subcortical limbic structures [82]. Magioncalda et al. proposed that BD was primarily characterized by immune-inflammatory factors causing white matter damage mainly encompassing the limbic network. Consequent phasic reconfigurations of intrinsic brain activity might superimpose onto structural damage in gray matter. The extent of gray matter damage could be related to cognitive deterioration that persist even during euthymia [83]. Specifically, the decreased organization of posterior white matter has been linked to impaired cognitive functions rather than emotional symptoms of BD [84]. White matter abnormality is a consistent finding in BD, as shown by several structural imaging studies since the 1990s and diffusion magnetic resonance imaging studies since the 2000s [85]. The longitudinal investigations measuring dynamics of functional connectivity across different mood states in the same patients, have shown that interoceptive sensorimotor activation pattern is more frequent during hypomania compared to other mood states, and is predictive for more severe symptoms of irritability and motor agitation. In contrast, a default-mode activation pattern is more frequent during depression compared to other mood states and compared to controls [86]. The white matter changes seem to represent a core and stable alteration in BD, while alterations in brain network balances seem to vary across the manic and depressive phases (Table 3).

**Table 3:** Studies exploring the cognition function with fMRI in bipolar disorder.

study	*N*	method	ROI/network	Main findings
Adler et al. (2004)	E = 15	Voxel-by-voxel	Whole brain	DLPFC/TC/BG/PPC ↑
	HS = 15			
Öngür et al. (2010)	BD = 17	ICA	DMN/mPFC	FC in the parietal cortex correlated with mania severity↑
	SCZ = 14			FC in the ventral mPFC↓
	HS = 15			FC in the mPFC (both SCZ and BD) ↓
Martino et al. (2016)	D = 20	SBA	DMN/SMN	DMN/SMN ratio correlated positively with clinical scores of depressive and negatively with manic symptoms
	M = 20			
	E = 20			
	HS = 40			
Wang et al. (2016)	BD = 37	Network analysis	DMN/LIM	FC in the DMN (bilateral medial PFC, left precuneus, right PCC) ↓
	HS = 37			FC right superior FG ↓
Robers et al. (2017)	BD = 49 FDR = 71 HS = 80	FC	IFG	BD: IFG-AI ↓ IFG-VLPFC ↓
McPhilemy et al. (2017)	BD = 35	RST	Frontal/parietal/cingulate/temporal/occipital regions	BD: response timing antisynchronous activity between regions of this subnetwork
	HS = 49			
Thomas et al. (2019)	BD = 42	ICA	CEN/LIM/SMN	BD: CEN-LIM ↑
	HS = 52	RST		CEN-SMN ↑
Tu et al. (2019)	BD = 100	FC	Thalamus/SN	salience network abnormalities in major psychiatric disorders
	MDD = 100			
	SCZ = 100			
	HS = 160			
Wang et al. (2020)	BD = 38	ICA	DMN/CEN/SN	BD: within the DMN ↓
	MDD = 35			BD: within the CEN ↓
	HS = 52			BD: inter-network CEN-SN ↑
Wang et al. (2020)	BD = 51	ICA	DMN/CEN/SN	BD/MDD: DMN-CEN ↓
	MDD = 51			
	HS = 21			
Liu et al. (2021)	E = 23	dFC	SMN/DMN	DMN and SMN can be used to distinguish depressed and euthymic states in BD
	D = 23			
	HS = 31			
Petersen et al. (2021)	BD = 153	ROI	DMN/CEN	DLPFC and parietal ↓
	HS = 52			DMN ↑

*BD* bipolar, *E* euthymic, *M* mania, *SCZ* schizophrenia, *D* depression, *MDD* major depression disorder, *HS* healthy subjects, *FC* functional connectivity, *ROI* region of interest, *RST* resting-state network, *dFC* dynamic functional connectivity, *SBA* seed-based analysis, *CCN* cognitive control network, *DLPFC* dorsolateral prefrontal cortex, *TC* temporal cortex, *BG* basal ganglia, *PPC* posterior parietal cortex, *DMN* default mode network, *SMN* sensorimotor network, *CEN* central executive network, *SN* salience network, *LIM* limbic system, *IFG* inferior frontal gyrus, *VLPFC* ventrolateral prefrontal cortex, *mPFC* medial prefrontal cortex.

## Treatments targeting cognition regulation network dysfunctions in BD

In the past several years, studies have explored the application of rTMS in BD utilizing resting state electroencephalography (EEG) data. Changes in resting state networks (RSNs) between pre and post rTMS were explored utilizing EEG functional network analysis, evaluated by exact low-resolution electromagnetic tomography. Bilateral stimulation of dlPFC produced changes in the activity of the SMN and resulted in significant changes in the executive functions, verbal memory, and depression symptoms [87]. One study exploring deep (H1-coil) rTMS interventions focused on the dlPFC for cognitive improvement in BD shows that the deep rTMS is a safe antidepressant intervention in bipolar patients, who usually present marked cognitive impairment. Another randomized, placebo-controlled, double-blind trial also revealed that no cognitive impairment was found for BD patients treated with transcranial direct current stimulation over the dlPFC [88]. The optimal brain area to be stimulated to promote cognitive enhancement is the critical issue [89].

The studies on the neural correlates of TMS-induced cognitive function have shown that fMRI-based neurofeedback targeted at dlPFC and superior frontal gyrus and suppression of activity in DMN regions like the hippocampus in which activity was correlated with working memory performance resulted in cognitive improvement [90, 91]. Miskowiak et al found that 8 weeks of treatment with erythropoietin (EPO) increased working memory capacity in UD and BD, which was accompanied by enhanced task-related activity in the right superior frontal gyrus and deactivation of the left hippocampus. Furthermore, Meusel et al. showed that cognitive remediation therapy increased task-related activity in lateral PFC, medial frontal gyrus, superior temporal, and lateral parietal regions. Similarly, Haldane et al. found that lamotrigine treatment in remitted BD patients increased working memory-related activation over time in bilateral superior frontal gyri, cingulate gyri, and left medial frontal gyrus, even in the absence of changes in performance [92]. A preliminary study in youth with BD reported that the patients with bipolar depression treated with lamotrigine had decreased amygdala activation while viewing negative stimuli as depressive symptoms improved [93]. The same research group reported that potentially less efficient baseline activation of left dlPFC and increased baseline activation of left vlPFC during an affective task predicted greater treatment response of quetiapine in youth BD patients [94]. This study provides a neural signature for bipolar patient and indicates that individuals with BD can show both hypoactivity in the dlPFC and prolonged activation of mPFC simultaneously, suggesting the dorsal and ventral prefrontal subregions as promising candidates for biological markers of treatment response in bipolar depression. A better understanding of how these brain regions respond to different treatments may help guide more personalized treatments for patients with BD.

To date, nonpharmacological approach has been implemented to remediate cognitive impairments among people with BD using computerized cognitive training [95]. The cognitive remediation (CR) could tackle trait-like neurocognitive deficits in various cognitive domains such as attention, executive functions, verbal memory and learning, and social cognition. One study investigating the neural correlates induced by CR using fMRI showed increased activation in the left hippocampus that correlated with improvements in a recollection memory task, as well as increased activations in lateral and medial prefrontal and lateral parietal regions that correlated with memory improvements in a 2-back memory task [91].

## Psychomotor alterations of neural network in BD

Although historically neglected, one of the most important findings in research of core behavioral features of BD is hyperactivity, reckless actions, impulsivity, and agitation during the manic phase, as well as physical and mental sluggishness, akinesia, volitional inhibition, and decreased activity levels during the depressive phase of illness. Manic and depressive episodes of BD show opposite psychomotor symptoms and the exact neurophysiological mechanism of psychomotor excitation and inhibition in mania and depression remain unclear. There are only a few MRI studies on the neural correlates of psychomotor phenomena. These psychomotor symptoms may depend on distinct patterns of alterations in the SMN, DMN, and CEN [9, 96, 97]. The SMN and related motor function are modulated by non-motor networks, such as DMN and CEN. The SMN mainly comprises the motor cortex (precentral gyrus, premotor and supplementary motor cortex) and middle cingulate cortex, somatosensory cortex (postcentral gyrus and parietal cortex), auditory cortex (superior temporal gyrus), as well as visual cortex (occipital cortex and associated dorsal parietal and inferior temporal areas), and is involved in exteroceptive awareness and motor activity [98]. The SMN is functionally connected with distinct regions of the thalamus, coherently with the structural connections, constituting segregated thalamo-cortical loops. The thalamo-cortical loops modulate the intrinsic activity and are directly connected with the external or internal environment, such that the sensory cortices receive inputs from the environment through the sensorial thalamic nuclei, while the motor and other specific cortices send outputs to the effector systems [99]. The thalamo-cortical loops are also connected with the basal ganglia and constitute different in-parallel cortico-striatopallido-thalamo-cortical loops. A recent study has reported that the BD patients show reduction in the cohesiveness of the SMN that likely disrupts the processing of sensorimotor information within the brain [100]. The alterations of interhemispheric dysfunction in SMN via corpus callosum using diffusion tensor imaging were reported in BD patients, which could be one of the pathophysiological bases of BD [101]. Other work has indicated that distinct changes within SMN and DMN during different phases of BD, i.e. predominant disruption of local connectivity within the SMN during depression, while disruption of both local and distant connectivity in the DMN during mania, consistent with the evidence showing an opposite pattern of alterations in mania and depression [102]. Recently, Martino and Magioncalda proposed a new theory that different combinations in neurotransmitter signaling favor network balancing into distinct functional brain states, which manifest in different combinations of excitation or inhibition in psychomotricity, affectivity, and thought, resulting in the manic, depressive, and mixed states of BD [13]. In this model, intrinsic brain activity is organized in distinct units in accordance with connectivity patterns and related setting of input/output processing, underlying different behavioral/phenomenological dimensions. An external unit (mainly involving the SMN) responds to the external environment and sets the exteroceptive input/somatomotor output processing, underlying the psychomotor dimension. An internal unit (mainly involving the SN) responds to the internal/body environment and sets the interoceptive input/visceromotor output processing, underlying the affective dimension. An index of intrinsic neuronal activity that favors the motor response to incoming stimuli, the more of neuronal variability in the SMN, the more excitation of psychomotor activity following cyclothymic temperament. Neuronal variability in SMN is higher in cyclothymic temperament and lower in depressive temperament [103]. The disbalance between SMN and DMN has been detected in BD, which a predominance of SMN occurs in mania along with greater global signal representation in SMN areas and reduced connectivity within the DMN [9]. Conversely, a predominance of DMN occurs in depression, as suggested by tilting in SMN/DMN balance toward the DMN at the expense of SMN [9, 104]. Northoff et al has proposed that the psychomotor mechanisms and their biochemical modulation were an example of a dimensional approach as suggested in RDoC and spatiotemporal psychopathology [19]. They identified three neural mechanisms: (i) serotoninergic modulation of dopamine-based subcortical–cortical motor circuit, (ii) reciprocal balances of DMN and sensory networks with SMN, and (iii) local synchronization of SMN with the brain’s global activity [19].

## Treatments targeting psychomotor symptoms in BD

The alterations in large-scale brain intrinsic activity, subcortical-cortical coupling, and neurotransmitters signaling, such as dopamine (DA) and serotonin (5-HT) have been independently detected in BD [9, 105, 106]. Based on the reported data, Conio et al. propose that the dopaminergic nigrostriatal and mesocorticolimbic pathway and serotonergic pathway modulate the balance of functional connectivity in SMN and DMN. The alterations in DA and 5-HT signaling lead to subcortical–cortical functional reorganization, which results in brain intrinsic activity (such as DMN and SMN) disbalancing, finally manifesting in manic or depressive states of BD [104]. Previous studies also demonstrate that altered DA and 5-HT transmission, which modulates activity of SMN and the DMN and balance between these networks, results in excitation or inhibition of affectivity, psychomotricity, and thought [5, 13]. The use of some DA antagonists and partial agonists to treat acute mania, bipolar depression and maintenance treatment has been proved by the Food and Drug Administration (FDA). It is likely that the altered DA neurotransmission caused by the ligands, which is associated with FC and activity in the SMN, SN, and DMN, improves psychomotor activity as well as affective-related functions, and contributes to the clinical efficacy of these drugs.

The psychomotor abnormalities are characterized by specific symptom pattern and constellation of motor, emotional, and cognitive symptoms [107, 108]. The essential intermediate unit between the prefrontal areas and limbic affective areas during emotion regulation is the supplementary motor area (SMA), which has been involved in stimulus reconceptualization and cognitive demands [26]. The study investigating anatomy and function of the somatosensory cortex in euthymic bipolar women has shown increased resting state functional connectivity between the right somatosensory cortex and the brain regions involved with affective regulation (insular cortex, inferior frontal gyrus and OFC) [109]. Chronic motor cortex stimulation has been reported as a treatment method for Parkinson’s disease and neuropathic pain [110, 111]. A recent case series highlights the efficacy of add-on high-definition transcranial direct current stimulation (HD-tDCS) over sham stimulation in alleviating obsessive compulsory disorder with comorbid BD when anodal stimulation of pre-SMA was provided with right orbitofrontal placement of cathode [112]. The data from a few studies suggest that tDCS stimulating the prefronto-cerebellar improve the impaired neurocognition, neurological soft signs, and sleep in BD during euthymia [113]. The improvement in sensory integration, motor sequencing and coordination, executive and visuospatial function after the tDCS session supports the hypothesis of prefronto-thalamic-cerebellar circuitry involvement in neurocognition with euthymic BD [114]. These stimulation methods (rTMS and tDCS) may be applied in SMN systems that the latter modulate subcortical–cortical motor circuits and thereby alleviate psychomotor symptoms.

## Conclusion

The neurobiological models that draw links between internal cognitive deficits, emotional processing, psychomotor activity dysfunction and neurocircuitry are beneficial because this approach meaningfully increases our understanding of BD with respect to clinical management. We propose a heuristic model of how to integrate information generated by recent neuroimaging studies into guiding principles toward treatments of BD. We hypothesized that (1) the core pathological alteration in BD is damage of fronto-limbic network that mainly results in emotional dysfunction; (2) that changes in intrinsic brain network, such as SMN, SN, DMN, CEN are associated with alterations of cognitive function; and (3) beyond the dopamine-driven basal ganglia-thalamo-cortical motor circuit modulated by other neurotransmitter systems such as 5-HT (subcortical–cortical modulation), the SMN and related motor function modulated by other non-motor networks such as the DMN are involved in psychomotor function. Although cognitive, emotional, and psychomotor domains are typically studied independently, basic research and emergent findings in BD suggest that there are important ties between cognitive deficits, the emotional and psychomotor disturbances observed in BD. Understanding these relationships is critical for elucidating relevant aspects related to functionality and vulnerability within BD and is essential for the development of novel treatment interventions. The following strategies based on neurocircuitry include: (1) enhancing PFC function to downregulate overactivated fronto-limbic activity via the engagement of top-down dorsal prefrontal regions, to alleviate impairments in emotion regulation; (2) modulating neurotransmitter transmission to balance the activities of excitatory and inhibitory neural networks involved in psychomotricity, affectivity and cognition; and (3) non-invasive neurostimulation in different cortical systems that modulate subcortical–cortical motor circuits to alleviate psychomotor symptoms. If the therapies of BD targeting specific circuit and corresponding clinical profiles, the treatment efficacy will be improved and guide clinical practice.
